# Multilocus sequence types of invasive pneumococcal isolates from Danish infants (0–90 days) 2003–2013

**DOI:** 10.1186/s13104-015-1540-y

**Published:** 2015-10-14

**Authors:** Hans-Christian Slotved, Tine Dalby, Steen Hoffmann

**Affiliations:** Neisseria and Streptococcus Reference Laboratory, Department of Microbiology and Infection Control, Statens Serum Institut, Artillerivej 5, 2300 Copenhagen, Denmark

**Keywords:** Infants, Invasive pneumococcal disease, IPD isolates, MLST, *Streptococcus pneumoniae*

## Abstract

**Background:**

A pneumococcal conjugate vaccine (PCV) has been part of the Danish childhood immunization programme since October 2007. It is administered at the ages of 3, 5 and 12 months and healthy infants younger than 90 days are consequently not vaccinated. Initially the PCV-7 vaccine was used but this was replaced by the PCV-13 in April 2010. Vaccination coverage in Denmark is approximately 90 %.The aim of this study was to present multilocus sequence typing (MLST) profiles of *Streptococcus pneumoniae* isolates from Danish infants (0–90 days) with invasive pneumococcal disease (IPD) in the period 2003–2013.

**Findings:**

32 IPD isolates were investigated for their MLST profiles. The identified sequence types (STs) had previously been observed in other European countries. Among the clones were ST 306 (serotype 1), ST 180 (serotype 3) and ST 191 (serotype 7F).

**Conclusions:**

The ST profile distribution in this study is similar to that observed in other European studies and show a variety of STs. Our data show that the majority of STs found in Denmark is also observed in other European countries, indicating that the IPD isolates were from clones generally circulating in Europe.

## Findings

Invasive pneumococcal disease (IPD) is one of the most frequent types of bacteraemia and meningitis in infants in Denmark as well as globally [1, 2]. With the introduction and use of the pneumococcal conjugate vaccines (PCV) in children, an effective protection has been provided against IPD caused by the serotypes included in the vaccines [3, 4]. The PCV-7 vaccine was introduced into the Danish childhood immunisation programme in October 2007, and in April 2010, it was replaced by a PCV-13 vaccine [5, 6]. Several studies have shown the effect of PCVs on the reduction of vaccine serotypes causing IPD [2, 5, 7].

The vaccination of children in Denmark starts at the age of 3 months; thus infants younger than 90 days are not vaccinated [8]. In a previous study [8], the serotype distribution for IPD cases from Danish infants (0–90 days) during the years 1943–2013 was described. Isolates from this study for the period 2003–2013 were tested for their clonal relationship using multi locus sequence typing (MLST). The present study aims at presenting a descriptive investigation on (1) whether the IPD isolates found in this group of children are dominated by a few clonal complexes or a variety of isolates consisting of singletons, and (2) if these IPD isolates belong to clones that circulate in Denmark only or are generally found in Europe.

Eligible for inclusion in the study were 44 IPD cases from the period 2003 to 2013. Detailed background information, e.g. inclusion criteria and pneumococcal identification details including phenotypic serotyping has previously been published [8]. Of the 44 cases, viable biological material was non-existent for 12 of them, all from before 2007. Thus, only 32 isolates could be analysed for their MLST profiles. The study is based on isolates from the national laboratory surveillance data on IPD. Since data and samples from patients were collected routinely for diagnostic and national surveillance purposes, no ethical approval or informed consents from parents or guardians were required. The study was approved by the Danish Data Protection Agency (record number 2007-41-0229).

The MLST analysis was performed at the Neisseria and Streptococcus Reference (NSR) laboratory at Statens Serum Institut (SSI) using a protocol previously described [9]. Briefly, the MLST primer pairs for *aroE*, *gdh*, *gki*, *recP*, *spi*, *xpt,* and *ddl* were used as described in the MLST online database (http://pubmlst.org/). All sequences were analysed using the BioNumerics software (Applied Maths, Sint-Martens-Latem, Belgium). Analysis and annotation of sequences for MLST were performed by the online tool at http://pubmlst.org/. From the allelic distance, groups of closely related isolates were identified. In this study, a clonal complex was defined as isolates sharing six of the seven loci that define the allelic profile, as defined by Feil et al. [10] and as used in recent studies [11, 12]. Clonal relationships based on the sharing of five out of the seven loci was also investigated. BioNumerics and eBURSTV3 software (http://pubmlst.org/) were used to establish the relationship among the isolates.

Table 1 presents the serotypes and MLST profiles of the 32 viable isolates. A variety of sequence types (STs) were found, and for some serotypes the same ST was observed over several years. eBURST analysis based on similarities in 6 out of 7 loci revealed that the STs were dominated by 16 singletons and 3 groups of related STs and that there were no predicted founder. Five of the nine serotype 7F isolates were ST 191, one was ST 1589 (which is related to ST 191, only one of the seven loci is different), and three were ST 1176. The two analysed serotype 1 isolates belonged to the same ST 306, one from 2004 and the other from 2011. A similar observation was also observed with two serotype 3 isolates which both were ST 180. Regarding non-vaccine types, the two serotype 6C isolates from 2008 and 2009 were ST 481 and ST 1692, respectively. Serotype 8 from 2003 was ST 53, and the serotype 8 isolate (ST 9761) from 2013 was clonally related to ST 53. The performance of a new eBURST analysis based on five out of the seven loci did not change the observations made when using six out of seven loci. The ST 177, ST 2731, ST 9761, ST 53, ST 191 and ST 1589 were found to be SLV, while all other STs in this study were singletons. No DLV or TLV were found.

**Table 1:** Pneumococcal serotypes and MLST profiles from Danish infants younger than 90 days with invasive pneumococcal disease from 2003 through 2013

Year	Number of isolates	PCV-7 serotypes (MLST-profiles in parentheses)	PCV-13 serotypes (MLST-profiles in parentheses)	Non-PCV serotypes (MLST-profiles in parentheses)
2003	6	23F (ST 440)	1^a^ 1^a^ 7F^a^ 7F^a^	8 (ST 53)
2004	4	19F (ST 177)	1 (ST 306) 3 (ST 180) 7F^a^	
2005	4	18C^a^ 18C^a^	1^a^	20^a^
2006	4	6B^a^	1^a^ 3^a^ 7F (ST 1176)	
October 2007: introduction of PCV-7 in the Danish childhood immunisation programme				
2007	5	18C (ST 1016) 19F (ST 2731) 19F (ST 177) 23F (ST 37)	19A (ST 199)	
2008	6	6B (ST 146) 19F (ST 3347)	7F (ST 191) 7F (ST 191)	6C (ST 481) 27 (ST 4676)
2009	3	18C (ST 113)	7F (ST 1589)	6C (ST 1692)
April 2010: introduction of PCV-13 in the Danish childhood immunisation programme				
2010	3		3 (ST 9107) 7F (ST 191) 19A (ST 199)	
2011	4		1 (ST 306) 3 (ST 180) 7F (ST 1176)	27 (ST 9760)
2012	1			12F (ST 989)
2013	4		1 (ST 306) 7F (ST 191) 7F (ST 1176)	8 (ST 9761)

^a^12 isolates from before 2007 were not available, so no STs could be established for these isolates

In the study by Lambertsen et al. [9] invasive penicillin non-susceptible *S. pneumoniae* from Denmark in the period from 2001 to 2005 were described. The study presented the MLST profiles of 43 isolates, showing that there were dominating clonal complexes, but also that the isolates showed many singletons. To the authors’ knowledge, this is the only other study describing the MLST distribution of invasive pneumococcal isolates in Denmark. It has to be considered that Lambertsen et al. [9] presented selected data (only penicillin non-susceptible isolates from before the PCV introduction in Denmark) while the present study presents data on isolates obtained after the PCV introduction and moreover only from infants. Hence, the two studies cannot be compared. The MLST profiles from the two studies are also very different, and only a few STs were either identical or clonally related between both studies, while the majority were different STs. 37 of the 43 tested isolates in the study by Lambertsen et al. [9] belonged to serotypes included in the PCV-7 and two belonged to the PCV-13 serotypes. Today, the circulation of serotypes included in the PCV-7 has been extensively reduced due to the impact of the vaccine. Moreover, since penicillin non-susceptibility is often associated with certain serotypes, the current study and the Lambertsen study would be expected to be very different with respect to serotypes as well as STs. The effect of the PCV-7 introduction in 2007 in Denmark [5] might be the reason why the PCV-7 serotypes found in the Lambertsen et al. study [9] are very seldomly observed today in Denmark [5].

The MLST profiles found in this study belong to well-known STs also found in other European countries [13–15]. Among these are ST 306 (serotype 1), ST 180 (serotype 3) and ST 191 (serotype 7F) (Table 1). Several of the STs reappeared over the years such as ST 306 (serotype 1) in 2004, 2011, and 2013, and ST 180 (serotype 3) in 2004 and 2011 (Table 1). This occurrence of STs over several years has also been described by Ardanuy et al. [13], who found ST 306 (serotype 1) and ST 180 (serotype 3) in 1999–2000 and again in 2007–2009. The observation that the STs in this study are generally seen in other European countries [13–15] may indicate that the majority of IPD isolates observed in unvaccinated Danish infants belong to strains that circulate generally in Europe and not only in Denmark. Two of the three non-vaccine serotypes [ST 9761 (serotype 8) and ST 989 (serotype 12F)] observed in 2011, 2012 and 2013 are clonally related (using the 6/7 loci definition) to serotypes in other European countries according to the *Streptococcus pneumoniae* isolates MLST database (Accessed 12 March 2015, http://pubmlst.org/). Apparently, the ST 9760 (serotype 27) strain observed in 2011 has only been found in Denmark previously, and is not clonally related to other serotypes using the 6/7 loci definition. To the authors’ knowledge, this ST has not been described elsewhere or recorded in other countries according to the MLST database (Accessed 20 July 2015, http://pubmlst.org/). Serotype 27 is rarely observed in Denmark, and since 2003 there has only been confirmed five IPD cases due to serotype 27.

In conclusion, the MLST profiles of IPD isolates from Danish unvaccinated infants younger than 90 days in this study are similar to the profiles observed in Europe. The isolates show a variety of STs that exceeds the number of different serotypes. There are, however, STs that reappear over the years from 2003 to 2013. The serotypes appearing after the PCV introduction are in general clones observed in other European countries, suggesting that future serotypes appearing in Denmark may well be clones already circulating in Europe, although domestic pneumococcal clones such as ST 9760 (serotype 27) also appear.

## Availability of supporting data

The dataset supporting the results of this article is included within the article.

## Abbreviations

DLV: double locus variant
IPD: invasive pneumococcal disease
MLST: multi locus sequence typing
PCV: pneumococcal conjugate vaccine
SLV: single locus variant
SSI: Statens Serum Institut
ST: sequence type
TLV: triple locus variants
